# Metal-Organic Frameworks: Synthetic Methods and Potential Applications

**DOI:** 10.3390/ma14020310

**Published:** 2021-01-09

**Authors:** Catherine P. Raptopoulou

**Affiliations:** Institute of Nanoscience and Nanotechnology, National Centre for Scientific Research “Demokritos”, 15310 Aghia Paraskevi, Attikis, Greece; c.raptopoulou@inn.demokritos.gr; Tel.: +30-210-650-3346

**Keywords:** metal-organic frameworks, coordination polymers, synthesis, applications

## Abstract

Metal-organic frameworks represent a porous class of materials that are build up from metal ions or oligonuclear metallic complexes and organic ligands. They can be considered as sub-class of coordination polymers and can be extended into one-dimension, two-dimensions, and three-dimensions. Depending on the size of the pores, MOFs are divided into nanoporous, mesoporous, and macroporous items. The latter two are usually amorphous. MOFs display high porosity, a large specific surface area, and high thermal stability due to the presence of coordination bonds. The pores can incorporate neutral molecules, such as solvent molecules, anions, and cations, depending on the overall charge of the MOF, gas molecules, and biomolecules. The structural diversity of the framework and the multifunctionality of the pores render this class of materials as candidates for a plethora of environmental and biomedical applications and also as catalysts, sensors, piezo/ferroelectric, thermoelectric, and magnetic materials. In the present review, the synthetic methods reported in the literature for preparing MOFs and their derived materials, and their potential applications in environment, energy, and biomedicine are discussed.

## 1. Introduction

Metal Organic Frameworks (MOFs) constitute a class of solid porous materials, which consist of metal ions or metallic clusters, which act as nodes, and polydentate organic ligands, which act as linkers between the nodes. The metal nodes (metal ions or metallic clusters) act as connection points and the organic ligands bridge the metal centers through coordination bonds, thus, forming networks of one-dimension, two-dimensions, or three-dimensions. The main structural features of the MOFs, which are directly related to their properties and applications, are the high porosity, the large volume of the pores, which can reach the 90% of the crystalline volume or more, the large specific surface area (several thousand m^2^·g^−1^), and the high thermal stability (250–500 °C) due to the presence of strong bonds (e.g., C–C, C–H, C–O, and M–O). An important sub-class of MOFs are the Isoreticular Metal Organic Frameworks (IRMOFs), which were first synthesized by the group of Yaghi [1]. The archetype IRMOF-1 was based on octahedral Zn-O-C clusters and 1,4-benzenedicarboxylic acid (BDC) bound to form a network with **pcu** topology. The series of IRMOFs retain the **pcu** topology and are based on varied organic linkers resulting in variable pore volumes and surface area. According to the terminology officially adopted by IUPAC on 2013 [2], MOFs are a sub-class of coordination networks (i.e., coordination compounds which extend to one-dimension, two-dimensions, or three-dimensions through repeating coordination entities), which are a sub-class of coordination polymers (i.e., coordination compounds with repeating coordination entities extending in 1, 2, or 3 dimensions, which do not need to be crystalline). MOFs are dynamic systems susceptible to structural changes upon external stimuli, such as temperature and pressure, and may not be crystalline.

The chemistry of MOFs has evolved rapidly in recent decades and it has become possible to adjust the size and shape of the pores, the network topology, and their surface area, so that the structures and properties of MOFs can be adapted to the needs of each application. The designed synthesis of new MOFs with desired physical and chemical properties in terms of Crystal Engineering principles requires the understanding of molecular and/or intermolecular interactions within the three-dimensional arrangement. The molecular interactions are the coordination bonds between the metal ions and the organic ligands, whereas the intermolecular interactions are the weak interactions such as hydrogen bonds, π–π interactions, etc. The designed synthesis of MOFs also requires complete control of the components and tools to be used, which is a fact that has been strongly argued by many researchers who believe that the complete control of all parameters of a chemical reaction is not feasible. On the other hand, lies the argument of the designed synthesis of IRMOFs, based on MOF-5, which resulted in a fairly large series of solids.

The structural characteristics of the MOFs are mainly affected by the large number of coordination geometries adopted by the metal ions and the use of oligonuclear metal clusters as nodes (secondary building units, SBUs), the geometrical characteristics, and the flexibility of the organic ligands, the role of the counterions, and the reaction solvent [3,4,5,6,7]. The network topology and dimensionality of the MOFs are strictly related to the different coordination geometries that can be adopted by the metal nodes, which vary depending on the electronic structure of the metal ions. Transition metal ions, especially those of the first row, lanthanides, and alkaline earth metals have been used because they display a wide variety of coordination numbers, geometries, and oxidation states, thus, offering synthetic and structural diversity. The use of rigid or flexible organic ligands plays a very important role in designing a MOF because the flexible ligands offer increased degrees of freedom with respect to the rigid ones and can lead to unpredictable crystal structures [8]. Organic molecules containing one or more N-donor or O-donor atoms are normally used as organic ligands to bridge between the metal ions in MOFs. Carboxylates (either aliphatic or aromatic containing one or more rings), pyridyl (e.g., pyrazine and 4.4′-bipyridyl derivatives) and cyano compounds, polyamines resulting from imidazole, oxalic acid, and benzene, phosphonates, sulfonates, and crown ethers are the most common ligands used. The anions counterbalance the positive charge of cationic MOFs and influence the supramolecular structure either being coordinated to the metal ions or by occupying the pores of the structure [9]. Organic cations can be hosted within the pores of anionic MOFs and can be exchanged by other cations, as in the case of bio-MOF-1, which hosts Me_2_NH_2_^+^ cations and solvate molecules and retains its crystallinity in solvent exchange experiments as well as during storage and release of cationic drug molecules [10]. Metal cations are usually coordinated to the original network, thus, altering its structural characteristics, whereas anionic metallic clusters require large porous structures and, in exceptional cases, can be exchanged by other cations for sensor applications [11]. The reaction solvent can affect the crystallization kinetics and the network topology through steric effects, fill coordination sites of the metal ions, complete the pores of the MOF, or participate in weak intermolecular interactions contributing to the crystal and thermal stability of the lattice.

The three-dimensional structure of MOFs is formed due to strong coordination bonds between the metal ions and the organic ligands and displays cavities and inner surfaces, which are occupied by counterions, guest molecules, and/or solvate molecules. Other types of interactions, such as hydrogen bonds, metal-metal bonds, and π–π interactions can occur and contribute to the stability of the MOFs. However, coordination bonds are stronger and provide more stable networks. Depending on the size of the cavities/pores, the MOFs are divided into nanoporous materials, with pores less than 20 Å in diameter, mesoporous materials (20–500 Å in diameter), and macroporous materials (over 500 Å in diameter). Most of the mesoporous and macroporous MOFs are amorphous materials [12]. Increasing the size of the pores is still a challenge since interpenetration prevents the presence of free empty space in a network. Interpenetration is favored by organic ligands with aromatic rings capable of the development of π–π interactions. Highly porous MOFs have been prepared by using poly-carboxylato and rigid alkyne-type ligands, as well as SBUs with large dimensions, which determine the size of the pores [13].

The present review presents the synthetic methods used to prepare MOFs of various dimensionality and porosity, and outlines their potential applications in the adsorption of many compounds, such as biologically important compounds (drugs, antibiotics, etc.), toxic pollutants and gas, electrochemical energy storage systems and sensors, catalysts and electrocatalysts, and efficient drug delivery carriers.

## 2. Synthesis of MOFs

**Figure 1:**
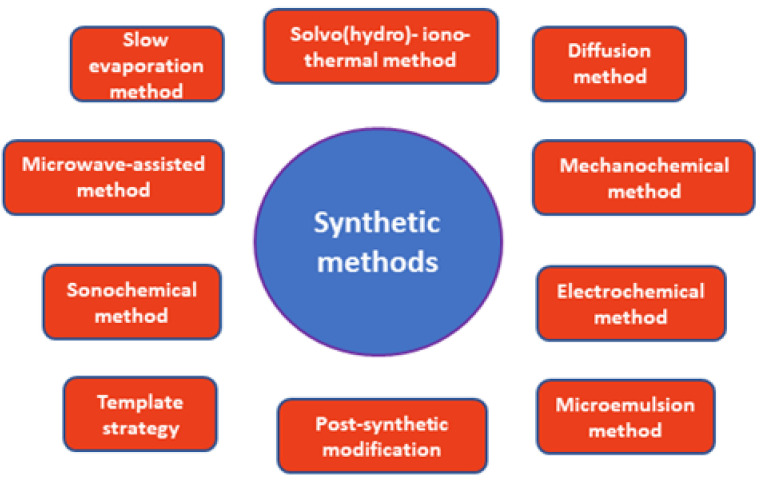
Synthetic methods used to obtain MOFs.

The synthesis of MOFs is determined by many factors related to the reaction time and temperature, the solvent used, the nature of the metal ions and the organic ligands, the size of the nodes and their structural characteristics, the presence of counterions, and the kinetics of the crystallization, which should lead to nucleation and crystal growth. In most cases, the synthesis of the MOFs is performed in the liquid phase by mixing solutions of the ligand and the metal salt. The choice of the solvent is based on its reactivity, solubility, and redox potential. The solvent also plays an important role in determining the thermodynamics and activation energy for each reaction. In some cases, solid state synthetic methods have been used even though difficulties in single crystal growth have been encountered. Slow evaporation of the reaction solution has been used very often to grow crystals of MOFs. In most cases, MOFs are synthesized under solvo(hydro)thermal conditions at a high temperature and pressure. This is the ‘classic’ method for preparing MOFs. Other alternative synthetic methods, such as mechanochemical, electrochemical, microwave, and sono-chemical methods, have been developed in recent years. These methods are low cost, faster, and yield cleaner products (Figure 1) [14,15].

### 2.1. Slow Evaporation and Diffusion Methods

Both methods are performed at room temperature and do not need energy supply. During the slow evaporation method, solutions of the reagents are mixed and left for slow evaporation and crystals are formed when a critical concentration is reached, to favor nucleation and crystal growth. Mixtures of low boiling point solvents are often used to accelerate the process [16,17]. During the diffusion method, solutions of the reagents are placed one on the top of the other, separated by a layer of solvent, or are gradually diffused by diving physical barriers. In some cases, gels are used as crystallization and diffusion media. Crystals are formed in the interface between the layers, after the gradual diffusion of the precipitate solvent into the separate layer [8]. The diffusion technique is used specifically if the products are not very soluble. MOF-5 or IRMOF-1 with formula [Zn_4_O(BDC)_3_]·(dmf)_8_(C_6_H_5_Cl)]_n_ (BDC^2−^ = 1,4-benzodicarboxylate) was prepared by diffusion of Et_3_N into a solution of Zn(NO_3_)_2_ and H_2_BDC in dmf/chlorobenzene and addition of a small amount of hydrogen peroxide to facilitate the formation of O^2−^ bind to the center of the SBU [18].

### 2.2. Solvo(Hydro)-Thermal and Iono-Thermal Method

Solvo(hydro)thermal reactions are carried out in closed vessels under autogenous pressure above the boiling point of the solvent [19]. Most of the MOFs reported so far, have been synthesized using solvo(hydro)thermal conditions [20,21,22,23,24]. The reactions are usually carried out in polar solvents using closed vessels (autoclaves) at temperatures in the range of 50–260 °C, and require long periods (hours and sometimes days). Teflon-lined autoclaves are used for reactions at high temperatures above 400 °C. The temperature of the reactions may be increased in order to facilitate bond formation, especially if kinetically inert ions are used, and to ensure proper crystallization. The temperature also affects the morphology of the crystals, while prolonged reaction times may lead to decomposition of the final product [25,26]. The cooling speed rate should be very slow and affects crystal growth. High boiling point solvents are most often used. The most common are dimethyl formamide (dmf), diethyl formamide (def), MeCN, MeOH, EtOH, H_2_O, Me_2_CO, or their mixtures. During solvo(hydro)thermal conditions, the initial reagents may undergo unexpected chemical transformations, which are not achieved under milder synthetic conditions, leading to new ligands formed in situ.

The ionothermal synthesis is based on the use of ionic liquids as solvents and templates and can be considered as a subclass of solvo(hydro)thermal methods. Ionic liquids are environmentally-friendly reagents, compared to conventional organic solvents, because of their low vapor pressure, high solubility for organic molecules, high thermal stability, and nonflammability, which makes them excellent reagents for the synthesis of MOFs as well as other classes of materials (i.e., zeolites and chalcogenides). Ionic liquids also offer both anions and cations as counterions and/or as templates for the frameworks of MOFs, and have been widely explored in recent years as alternatives for the synthesis of MOFs [27].

### 2.3. Microwave-Assisted Method

The method is often used for the synthesis of organic and nanoporous inorganic materials [28]. More recently, the method was used for the synthesis of metal clusters [29] and MOFs [30,31]. The advantages of the method are the short reaction time required, the high yield, and the low cost. The microwave-assisted synthesis of HKUST-1 with formula [Cu_3_(BTC)_2_(H_2_O)_3_] (BTC^3−^ = 1,3,5-benzenetricarboxylate) gave crystals with improved yield and physical properties, requiring a much shorter reaction time, with respect to its conventional hydrothermal synthesis [32]. Despite the fact that the specific technique cannot produce crystals, the microwaves facilitate the motion of the molecules, leading to nucleation and formation of crystals with a controlled shape and size by appropriately adjusting the concentration and the temperature of the reaction [33].

### 2.4. Mechanochemical Method

The method uses mechanical forces, instead of using a solvent, at room temperature, to form coordination bonds by either manual grinding of the reagents or more often in automatic ball mills. In some cases, a small amount of solvent may be added into the solid reaction mixture and succeeded to obtain one-dimensional, two-dimensional, and three-dimensional coordination polymers [34]. The mechanochemical method facilitates mass transfer, reduces particle size, heats, and locally melts the reagents, thus, accelerating the reaction time. It constitutes an environmentally-friendly green chemistry method, which produces materials of high purity and high efficiency at short reaction times [35]. The application of mechanical chemistry to the synthesis of MOFs is additionally attractive because it is an alternative to the high temperature and pressure solvo(hydro)thermal synthesis. The biggest disadvantage of the method is the isolation of amorphous products, unsuitable for single-crystal X-ray structural studies.

### 2.5. Electrochemical Method

The method is used for the synthesis of MOF powders on an industrial scale. The metal ion is provided by anodic dissolution into reaction mixtures that contain the organic ligands and electrolytes. The major advantages of this method are the slighter temperatures of reaction and extremely quick synthesis under milder conditions, compared to solvothermal method. Several MOFs, such as HKUST-1, ZIF-8, MIL-100(Al), MIL-53(Al), and NH_2_-MIL-53(Al), have been synthesized by this method in an electrochemical cell, and the influence of several reaction parameters on their yield and texture properties have been investigated [36].

### 2.6. Sonochemical Method

Sonochemistry deals with the chemical transformations of molecules under high-energy ultrasonic radiation (20 kHz–10 MHz). The bubbles formed when a reaction solution is irradiated with ultrasound radiation create local hot spots of a short lifetime with a high temperature and pressure, which promote chemical reactions and immediate formation of crystallization nuclei [37,38,39]. High quality crystals of MOF-5 and MOF-177 with a size of 5–25 μm and 5–20 μm, respectively, were prepared via the sono-chemical method in the presence of 1-methyl-2-pyrrolidone as a solvent, in a substantially reduced reaction time [40,41].

### 2.7. Microemulsion Method

The method is widely used in the preparation of nanoparticles and has recently been used to synthesize MOFs [42,43]. Water microemulsions contain nanometer-sized water droplets immobilized by a surfactant on the organic phase. The micelles of the microemulsions act as nanoreactors and control the kinetics of nucleation and crystal growth. The size and number of micelles in the microemulsion can be adjusted by varying the water to surfactant ratio and the type of the surfactant. The method is advantageous because the dimensions of the nanoscale materials can be controlled, while the major disadvantages are the high cost and the fact that most of the surfactants used are environmental pollutants.

### 2.8. Post-Synthetic Modification

The method involves the introduction of desired functional groups into the MOFs after their synthesis (PSM, Post Synthetic Modification) and is essentially a process of chemical transformation of the MOFs after their isolation. The method has been widely used to prepare isostructural MOFs with different physical and chemical properties [44,45,46,47]. For example, IRMOF-3 containing 2-amino-1,4-benzenedicarboxylic acid can undergo chemical modification with a diverse series of anhydrides and isocyanates yielding isostructural MOFs containing different functional groups [48]. Post-synthetic modification can involve the replacement of the primary structural units of the MOFs (BBR, Building Block Replacement), including solvent-assisted ligand exchange (SALE, Solvent-assisted Linker Exchange) [49], replacement of the non-bridging ligands, and metal nodes. Complete exchange of the organic ligands can occur during the SALE process, adding different functionality to the MOF. BBR reactions involve the heterogeneous exchange of ligands or metal ions by breaking and forming chemical bonds within the original MOF [50]. BBR methods are used when the direct synthesis of the desired MOF is not achieved, functionalizing the pores or nodes within the MOFs, affording or enhancing desired functional properties such as catalysis, selective gas adsorption, redox, and ionic conductivity. BBR reactions are observed only on or near the outer surface of MOF crystals [51,52,53]. Post-synthetic modification reactions can create defects on the MOFs either by missing or replacing metal nodes or by missing or partially replaced organic linkers. Such defects can be also generated during the conventional synthesis of MOFs and during the crystallization process and crystal growth [54]. Mixed-metal MOFs, containing at least two metal ions in their framework, can be prepared under post-synthetic methods, as well as from one-pot methods or by using metalloligands, and possess new properties and activities due to the presence of the second metal ion [55]. Structural defects and inhomogeneities are often related to important material properties, and, hence, defect engineering has been effectively applied in order to modify and functionalize MOFs for applications in catalysis, gas sorption, separation and storage, and luminescent and magnetic materials.

### 2.9. Template Strategies

The use of template molecules in the reaction mixture can lead to novel MOFs, which are difficult to obtain by traditional synthetic methods [56]. The template molecules that have been widely used are small organic molecules, including organic solvents, organic amines, carboxylic acids, N-heterocyclic aromatic compounds, ion liquids, surfactants, and other organic molecules. Each of this class of organic compounds affects the synthesis and crystallization of the MOF differently. For example, the solvent polarity and solubility affect the crystallization of the MOF, the organic amines adjust the pH of the reaction solution and facilitate the deprotonation of the organic ligands, carboxylate compounds act as ligands to the metal centers and can fill the pores of the MOF, aromatic heterocyclic compounds act as counterions when protonated and as weak organic bases, ion liquids act as solvents and counterions, and surfactants form micelles in solvents, which determine the shape and size of the MOFs. Other molecules, which may act as templates, are coordination compounds (e.g., [Ru(2,2′-bipy)_3_]^2+^), polyoxometalates, block co-polymers, MOFs, polystyrene spheres, substrates such as graphene oxide, and rarely biomacromolecules. The template synthesis strategy is used for the preparation of hierarchical porous materials, with mesoporous and microporous channels for hosting large molecules such as proteins and enzymes. However, the most common synthetic approach for hierarchical MOFs is the reticular chemistry strategy by using ligands of extending length to obtain MOFs of the same topology but with a variable pore size.

## 3. Applications of MOFs

MOFs display a range of structural features, namely large surface area, high porosity, crystallinity and thermal stability, and functionality of pores and frameworks, which render them promising materials for environmental and biomedical applications, as catalysts, sensors, absorbers for toxic gases, and metal ions.

### 3.1. Gas Adsorption/Separation/Storage for Energy and Environmental Applications

MOFs have been extensively studied for applications in gas storage. For example, H_2_ and CH_4_ represent alternative energy resources for future vehicles, and their effective usage still remains a challenge for the automotive industry. The capture of toxic industrial gases, such as NH_3_ and H_2_S, and volatile hydrocarbons, like benzene, as well as the removal of SO_2_ and NO_x_ from flue gas, are of great importance for environmental protection. A very critical step in the chemical industry is the separation of mixtures of gases, such as CO_2_ capture and CO_2_/CH_4_, CO_2_/N_2_ separation, O_2_ purification, and so on.

**Figure 2:**
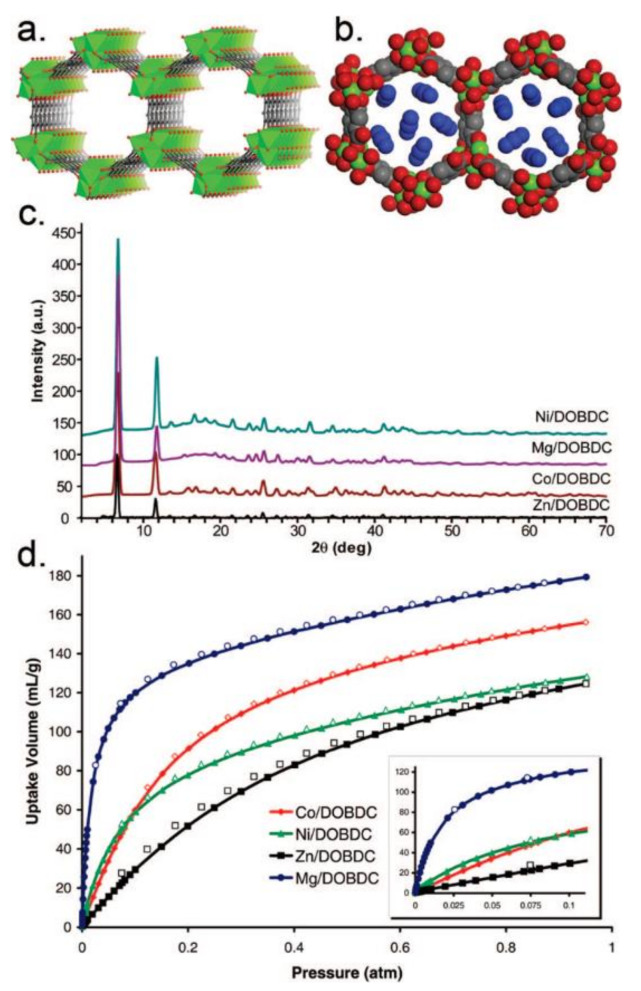
(**a**) The 1D channels of MOF-74-Mg, Mg-DOBDC (solvents omitted). (**b**) Space-filling model of the pore structure of MOF-74-Mg (Mg, green. C, grey. O, red). (**c**) PXRD data for the isostructural MOFs M-DOBDC (M = Zn, Co, Mg, Ni). (**d**) CO_2_ sorption isotherm (296K, 0 to 1 atm) of the isostructural MOFs M-DOBDC (inset: CO_2_ sorption isotherms at 296 K, 0 to 0.1 atm). Reprinted with permission from J. Am. Chem. Soc. 2008, 130, 10870–10871 ([57]). Copyright 2008 American Chemical Society.

CO_2_ is the main greenhouse gas and is responsible for global warming and for water acidification. MOF-74-Mg, which is the magnesium analogue of MOF-74, shows the highest CO_2_ uptake capacity of 228 and 180 cm^3^·g^−1^ at 273 and 298 K and 1 bar, respectively (Figure 2) [57]. The exceptional CO_2_ uptake by MOF-74-Mg is attributed to the increased ionic character of the Mg-O bond, which imparts additional uptake beyond weight effects while maintaining the reversibility of adsorption. MOF-210 has a very high surface area of 10,450 m^2^·g^−1^ and shows a CO_2_ uptake value of 2400 mg·g^−1^ (74.2 wt %, 50 bar at 298 K), which is larger than that of MOF-177 or MIL-101(Cr) (60 wt % and 56.9 wt %, respectively) [58,59,60]. MOF-200 has a similar CO_2_ uptake as MOF-210 under similar conditions. Other MOFs, which show considerably higher CO_2_ uptake compared with other solid materials, are the NU-100 (69.8 wt %, 40 bar at 298 K), the MOF-5 (58 wt %, 10 bar at 273 K), and the HKUST-1 (19.8 wt %, 1 bar at 298 K).

Synthetic strategies for the preparation of MOFs with efficient CO_2_ uptake capacity have been developed and include amine incorporation, introduction of functional groups, and additional metal ions and control of pore size. The method used in the industry for CO_2_ separation is the amine scrubbing, which is high-energy consuming and presents disadvantages, such as amine degradation and equipment corrosion. Alternatively, MOFs incorporating amines have been examined as potential candidates for CO_2_ separation. For example, incorporation of *N*,*N*′-dimethylethylenediamine (mmen) within the [Mg_2_(dobpdc)] MOF (dobpdc^4−^ = 4,4′-dioxido-3,3′-biphenyldicarboxylate) afforded [mmen-Mg_2_(dobpdc)], which displays an exceptional capacity for CO_2_ adsorption at low pressures, 2.0 mmol·g^−1^ (8.1 wt %) at 0.39 mbar, and 25 °C and 3.14 mmol·g^−1^ (12.1 wt %) at 0.15 bar and 40 °C, at conditions relevant to removal of CO_2_ from air and flue gas, respectively [61]. In addition, [en-Mg_2_(dobpdc)] and [dmen-Mg_2_(dobpdc)] (en = ethylenediamine, dmen = *N*,*N*′-dimethylethylenediamine) display significant CO_2_ uptakes (3.63 mmol·g^−1^ and 3.77 mmol·g^−1^, respectively) at 0.15 bar [62]. Bio-MOF-11, [Co_2_(ad)_2_(CH_3_CO_2_)_2_]·2dmf·0.5H_2_O (ad^−^ = adeninate) contains pyrimidine and amino groups within the pores of the framework and exhibits high CO_2_ capacity (~6 mmol·g^−1^ at 273 K) and exceptional selectivity for CO_2_ over N_2_ at 273 K (81:1) and 298 K (75:1) [63]. MOFs functionalized with low-molecular weight polymers containing amino groups, such as PEI (polyethyleneimine), have shown impressive CO_2_ uptake, which were many times larger than the respective MOFs. For example, PEI-MIL-101-125 display CO_2_ uptake of 3.95 and 4.51 mmol·g^−1^, respectively, over four times than that of MIL-101-125, and PEI@UiO-66 shows CO_2_ uptake up to 1.65 mmol·g^−1^ and CO_2_/CH_4_ selectivity 111, which are much larger values than UiO-66 [64,65]. Yaghi and co-workers reported the functionalization of the organic ligand of IRMOF-74-III with primary amine through ligand modification, thus, yielding six analogs with different functional groups (–CH_3_, –NH_2_, –CH_2_NHBoc, –CH_2_NMeBoc, –CH_2_NH_2_, and –CH_2_NHMe). Spectroscopic data revealed that CO_2_ binds chemically to IRMOF-74-III-CH_2_NH_2_ and IRMOF-74-III-CH_2_NHMe to form carbamic species. The CO_2_ uptake of IRMOF-74-III-CH_2_NH_2_ is 3.2 mmol·g^−1^ at 800 Torr and 298 K [66]. Introduction of polar functional groups in the pores of MOFs through direct synthesis or post-synthesis modification was proved an efficient method to enhance the adsorption capacity and selectivity of CO_2_. For example, UPC-12 exhibits high selectivity for CO_2_ due to the formation of H-bonds between the CO_2_ molecules and the -COOH groups within the pores and the π–π stacking interactions between the CO_2_ molecules and the bpy moieties of the MOF [67]. NbO-type MOFs retain the NbO-type structure upon functionalization with different groups, such as amide, nitro groups, and N-heterocycles, and display higher CO_2_ uptake than the parent MOFs [68,69,70,71,72]. Ligand functionalized UiO-66, UiO-66(Zr)-(COOH)_2_ shows high CO_2_/N_2_ selectivity of 56 upon a 15/85 CO_2_/N_2_ gas mixture at 303 K and 1 bar, and UiO-67 functionalized BUT-10 and BUT-11 show enhanced CO_2_ adsorption uptakes (50.6 and 53.5 cm^3^·g^−1^, respectively) and separation selectivity over N_2_ and CH_4_ (18.6 and 31.5 for a 15/85 CO_2_/N_2_ gas mixture, and 5.1 and 9.0 for a 10/90 CO_2_/CH_4_ gas mixture) [73,74]. The control of the pore size of the MOFs allows inclusion of smaller guests (e.g., CO_2_ 3.30 Å) and enables ultra-high selectivity. However, the precise control of pores with a size of 3–4 Å is very difficult. Examples of isoreticular MOFs, SIFSIX-2-Cu, SIFSIX-2-Cu-i, SIFSIX-3-Zn, and SIFSIX-3-Cu showed more efficient CO_2_ capture for the latter, which exhibits the smallest pore size [75,76]. The approach of ‘Single Molecule Trap’ (SMT) for the capture of a single CO_2_ molecule was developed by Zhou and co-workers who prepared paddlewheel dicopper complexes (SMT-1-2) with intramolecular metal-metal distance of 7.4 Å, suitable for the accommodation of one CO_2_ molecule. Incorporation of SMT-1 to the 3D framework of PCN-88 enhanced the CO_2_ uptake to 4.20 mmol·g^−1^ at 296 K and 1 bar with respect to 0.63 mmol·g^−1^ for SMT-1 under identical conditions [77]. MOFs based on heterometallic SBUs of the general formula [M^II^_2_M^III^(OH)(COO)_6_], CPM-200s, display excellent CO_2_ uptake capacity, 207.6 and 190.9 cm^3^·g^−1^ for CPM-200-Fe/Mg and CPM-200-In/Mg, respectively, at 273 K and 1 bar, comparable to the value of MOF-74-Mg [78]. Other examples of heterometallic MOFs such as MIL-101(Cr,Mg) and UiO-66(Li,Na,K,Pb) display higher CO_2_ uptake with respect to the parent MOF, but is much smaller than MOF-74-Mg and CPM-200s, which hold the highest values [79,80,81,82]. The mixed-metal solid-solution MOFs [(AlOH)_1−x_(VO)_x_L] (*x* = 0–1, L = 1,4-benzenedicarboxylate) display a total uptake of 11–14 mmol·g^−1^ at 1 bar for the low vanadium content species, which is comparable with the values found for other members of the MIL-53 family [83].

Light hydrocarbon separation is a very important and crucial process in the petroleum industry, and their efficient separation will reduce the energy consumption and cost. Ethylene (C_2_H_4_) and propylene (C_3_H_6_) are used in the production of polymers. During the production of C_2_H_4_, an impurity of ~1% of C_2_H_2_ is also produced. The microporous MOF [Cu(atbdc)] (H_2_atbdc = 5-(5-amino-1*H*-tetrazol-1-yl)-1.3-benzenedicarboxylic acid), UTSA-100, displays high C_2_H_2_/C_2_H_4_ selectivity and high C_2_H_2_ uptake from mixtures containing 1% acetylene. At 296 K and 1 atm, the acetylene and ethylene uptake amount of UTSA-100 are 95.6 and 37.2 cm^3^·g^−1^, respectively, which is much higher than that of M’MOF-3 [84,85]. Other examples of MOFs exhibiting high C_2_H_2_ uptake are UTSA-300 (3.41 mmol·g^−1^ at 273 K and 1 bar) [86], SIFSIX-1-Cu (8.50 mmol·g^−1^ at 298 K and 1 bar) [87], [Mn_3_(bipy)_3_(H_2_O)_4_][Mn(CN)_6_]·2(bipy)·4H_2_O (3.2 mmol·g^−1^ at 27–83 K and 1 bar) [88], and NOTT-300 (6.34 mmol·g^−1^ at 293 K and 1 bar) [89]. In addition, the raw propylene (C_3_H_6_) product contains trace impurity of propyne (C_3_H_4_), which is highly undesirable. Chen and co-workers reported a flexible-robust MOF, ELM-12, which shows strong binding affinity and suitable pore confinement for propyne, and obtained propylene with a purity over 99.9998%, which is the propyne impurity removed to a concentration below 2 ppm [90]. The separation of C_2_H_4_ and C_3_H_6_ from their mixtures with the respective alkanes by using distillation processes shows low efficiency because of the similarity of their boiling points. Their separation can be alternatively achieved by the formation of π-complexes of olefins with transition metal cations [91,92], and by using MOFs, such as KAUST-7, which contains channels allowing the adsorption of C_3_H_6_ but does not permit the C_3_H_8_ to diffuse/adsorb into the pore system [93,94]. Another important and difficult process in the chemical industry is the separation of benzene and cyclohexane, as well as C_2_H_2_/CO_2_ separation. Conventional distillation is high energy consuming. Therefore, alternative methods involving the use of suitable MOFs containing open metal sites, or introducing π–π stacking interactions of the π-electron deficient pore surface and π-rich guest molecules have been developed [95,96,97,98].

Alternative technologies for H_2_ storage and its potential use as renewable fuel for vehicle applications have long explored during the early years of MOFs development. The required materials should meet the U.S. Department of Energy (DOE) requirements, which are 5.5 wt % H_2_ gravimetric capacity achievable in the temperature range of −40 to 60 °C with a maximum pressure of 100 bar. Well-known MOFs, such as MOF-5, MOF-177, and UiO-66 display H_2_ uptake 5 wt % (77 K, 90 bar), 7.5 wt % (77 K, 80 bar), and 4.2 wt % (77 K, 60 bar), respectively [99,100,101]. Recent advances include the synthesis of a series of Pd-doped MIL-101 samples with different Pd content affecting their H_2_ storage performances, Pt nanoparticles on the outer surface of MOF-5, and then coating with hydrophobic microporous carbon black (CB/Pt/MOF-5 composite), which displays 41% higher H_2_ uptake than that of MOF-5, incorporation of polarized organic units into MOFs to improve the binding energy of H_2_ (e.g., MOF-649 and MOF-650 with internally polarized 2,6-azulenedicarboxylate), and carbon nanodots functional MOFs, Cdots@UMCM-1a, with efficiently enhanced H_2_ storage capacity attributed to specific interactions between the H_2_ adsorbate and polar groups on the surface of the Cdots [102,103,104,105].

**Figure 3:**
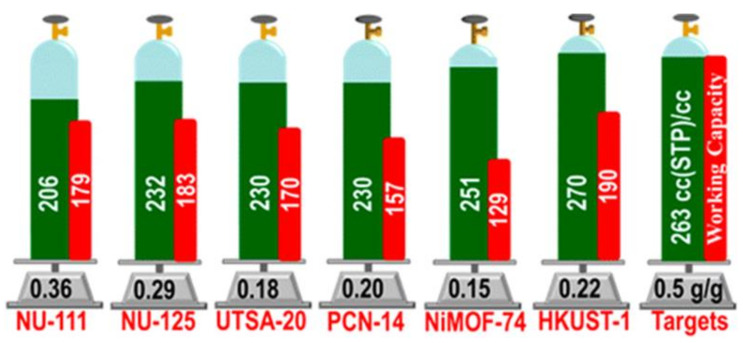
Methane uptake properties (green) and working capacity (red) of selected MOFs. Reprinted with permission from J. Am. Chem. Soc. 2013, 135, 11887–11894 ([107]). Copyright 2013 American Chemical Society.

Great progress has been made during recent decades on MOFs for applications on storage of CH_4_, which is the primary component of natural gas. For vehicle applications, an ideal MOF should have both high CH_4_ storage capacity and high CH_4_ deliverable capacity. Promising examples, which meet the volumetric target set by DOE (263 cm^3^·cm^−3^ at 35 bar), are HKUST-1 with total uptake 267 and 272 cm^3^·cm^−3^ at 65 and 80 bar, respectively, and working capacity of 190 and 200 cm^3^·cm^−3^ at 65 and 80 bar, respectively, UTSA-76 with total uptake of 257 cm^3^·cm^−3^ at 65 bar, and working capacity of 197 cm^3^·cm^−3^ at 80 bar, Ni-MOF-74 with total uptake of 251 and 267 cm^3^·cm^−3^ at 65 and 80 bar, respectively, and working capacity of 129 and 152 cm^3^·cm^−3^ at 65 and 80 bar, respectively, NJZU-53 with total uptake 241 cm^3^·cm^−3^ at 65 bar, and working capacity of 190 cm^3^·cm^−3^ at 65 bar, PCN-14 with total uptake of 230 and 250 cm^3^·cm^−3^ at 65 and 80 bar, respectively, and working capacity of 157 and 178 cm^3^·cm^−3^ at 65 and 80 bar, respectively, and MOF-519 with total uptake of 279 cm^3^·cm^−3^ at 80 bar, and working capacity of 230 cm^3^·cm^−3^ at 80 bar (Figure 3) [106,107,108,109].

MOFs have also been examined for the removal of hazard and toxic species produced via coal combustion and refinery processes, such as CO, NH_3_, NO_2_, SO_2_, H_2_S, benzene, etc. Besides their toxic pollutant character, these materials are important in the chemical industry as sources for the production of commodity chemicals. A Cu(I) loaded MOF, Cu(I)@MIL-100(Fe) shows CO adsorption capacity of 2.78 mmol·g^−1^ at 298 K and 1 bar, about seven times than that of MIL-100(Fe), and CO/N_2_ adsorption selectivity of 169, due to strong π-complexation between Cu(I)@MIL-100(Fe) and CO [110]. Defect-engineered MOFs [Ru_3_(btc)_2−x_(pydc)_x_X_y_] (X = Cl, OH, OAc; *x* = 0.1, 0.2, 0.6, 1.0; 0 ≤ *y* ≤ 1.5, H_3_btc = benzene-1,3,5-tricarboxylic acid; H_2_pydc = pyridine-3,5-dicarboxylic acid) derived by incorporation of organic ligand H_2_pydc into the framework of the mixed-valence Ru^II/III^ MOF [Ru_3_(btc)_2_Cl_1_._5_], display CO total uptake up to 3.88 mmol·g^−1^, which is 2–3 times larger than that of the parent MOF [111]. NH_3_ is among the industrial chemicals of highest toxicity and the development of materials for its adsorptive removal from air is of high importance. Dincă and co-workers reported the MOFs, [M_2_Cl_2_(BTDD)(H_2_O)_2_] (M = Mn, Ni, Co, BTDD = bis(1*H*-1,2,3-triazolo [4,5-b],[4′,5′-*i*]dibenzo-dioxin), which display NH_3_ uptake of 15.47, 12.00, and 12.02 mmol·g^−1^ at 298 K and 1 bar [112]. Lan and co-workers reported the removal of carcinogenic benzene by the isoreticular MOFs, NENU-511, NENU-512, NENU-513, and NENU-514 with uptake of 1556, 1519, 1687, and 1311 mg·g^−1^, respectively [113]. Zr(IV)-based MOFs, such as UiO-66-NH_2_, urea modified UiO-66 and UiO-66-ox have been widely investigated for the removal of NO_2_ due to their high chemical stability [114,115,116]. [M(btc)(ted)_0.5_] (M = Ni, Zn, bdc = 1,4-benzenedicarboxylate, ted = triethylenediamine), NOT-202a, MFM-300(In), and SIFSIX-1-Cu are among the MOFs examined for the removal of residual SO_2_ in flue gas, a process of fundamental importance because traces of SO_2_ (500–3000 ppm) are produced by coal combustion along with CO_2_ (10–12%), and react with the organic amines used during the removal of CO_2_ with the scrubbing process, thus, causing permanent loss of amine activity and decreasing the efficiency of the process [117,118,119,120]. The removal of H_2_S from the refinery-off gases and natural gas is necessary in order to avoid poisoning of the gases and the catalyst involved in the subsequent utilization of H_2_ and CH_4_. Prominent examples of MOFs proposed for H_2_S removal are Ga-soc-MOF, rare earth-based MOFs with **fcu** topology, kag-MOF-1, and composites containing Cu-BTC and S-doped or N-doped graphite oxides [121,122,123,124].

### 3.2. Sensing Applications

MOFs are especially attractive as novel sensing materials because they display a high surface area, which enhances detective sensitivity, specific structural features (open metal sites, tunable pore sizes, etc.), which promote host-guest interactions and selectivity, and flexible porosity, which enables reversible release and uptake of small molecules, cations and anions, biomolecules, and so on. The guest molecules can induce visible changes including a shift of the emission spectrum or change in the emitting color, and change in the fluorescence intensity such as ‘turn-on’ and ‘turn-off’ processes.

A thermostable Mg-based MOF, [Mg(pdda)(dmf)] (H_2_pdda = 4,4′-(pyrazine-2,6-diyl) dibenzoic acid), which contains nanoholes and non-coordinating nitrogen atoms inside the walls of the holes, displays high selectivity for Eu^3+^ ions at low concentrations in aqueous solutions [125]. A luminescent Ln-MOF, [Me_2_NH_2_][Tb(bptc)] (H_4_bptc = 3,3′,5,5′-tetracarboxylic acid) exhibits rare chiral helical channels despite the achiral nature of the organic ligand. Luminescent studies showed highly selective fluorescence quenching response to Fe^3+^ ions in a liquid suspension, rendering it as a potential chemo-sensor for Fe^3+^ ions [126]. A bimetallic Eu-Tb MOF with 1,4-benzenedicarboxylate ligands showed Pb^2+^ selectivity in polluted environmental waters. The color of the luminescent Ln-MOFs could be fine-tuned from green to red by doping the MOFs with different Tb/Eu ratios, and, in the presence of Pb^2+^, the emission color of the MOFs changes from red-orange to green, which is visually observed by naked eyes [127]. A Cd-MOF, [Cd(edda)] (H_4_edda = 5,5′-ethane-1,2-diylbis(oxy)]diisophthalic acid), exhibits ratiometric fluorescence response to Hg^2+^ for the first time with a fast response (~15 s) and especially high sensitivity of ~2 nM below the permissible limits in drinking water set by the U.S. Environmental Protection Agency. This behavior is attributed to the collapse of the crystal structure of the Cd-MOF induced by Hg^2+^ [128]. MOFs have been proven as very promising materials for the uranium extraction during radionuclide separation and seawater mining due to their ability for post grafting with functional groups with strong affinity for the uranium ions and porous functionalization for storage of hydrated U(IV) ions. HKUST-1, UiO-66, MILs, and ZIF-8 display stability under gamma irradiation. The phosphoryl-urea-functionalized UiO-68(Zr) MOF was the first organo-modified MOF that exhibited uranium extraction behavior. Examples of phosphonate-functionalized, amidoxime-functionalized, amine-functionalized MOFs, among others, display adsorption capacity up to 360 mg·g^−1^ [129].

Volatile organic molecules and explosive compounds can be efficiently detected by MOFs based on guest-dependent luminescent responses either by shifting of the emission spectrum or by changes in the luminescent intensity [130,131,132].

**Figure 4:**
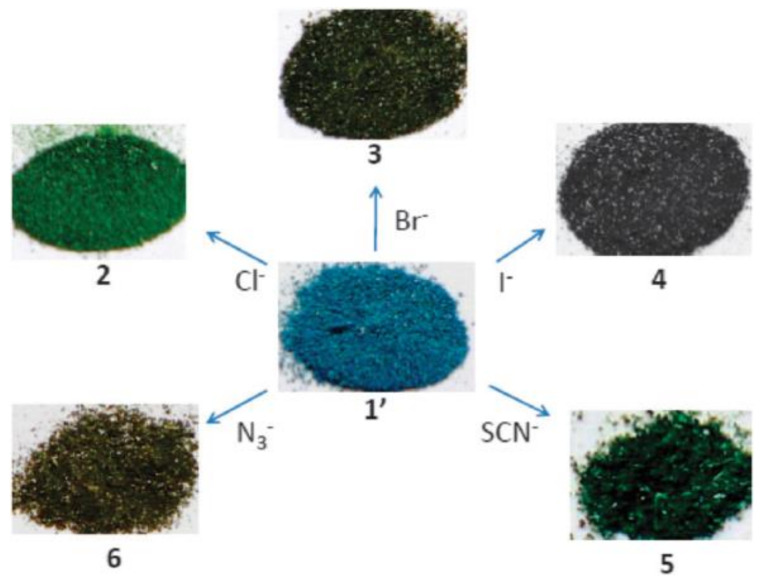
Color change of [CuL_2_(H_2_O)_0_._5_](NO_3_)_2_ (**1′**) upon exchange of nitrates by the indicated anions. Chem. Commun. 2012, 48, 2946–2948 ([139])—Reproduced by permission from The Royal Society of Chemistry.

Various anions have been successfully detected by MOF-based sensors. [Ln_2_(bpdc)(bdc)_2_(H_2_O)_2_] (Ln = Eu, Tb; H_2_bpdc = 2,2′-bipyridine-3,3′-dicarboxylic acid) can detect F^−^ ions based on a significant decrease in fluorescence, and [Ln_2_Zn(L)_3_(H_2_O)_4_](NO_3_)_2_ (Ln = Eu, Tb; L = 4,4′-dicarboxylate-2,2′-dipyridine anion) shows high selectivity and sensitivity to I^−^ ions [133,134]. An Ln-mucinate MOF shows CO_3_^2−^ sensing ability through the greatest luminescence enhancement over other anions, and MOF-based thin-film show selectivity on CO_3_^2−^ over other anions, e.g., SO_4_^2−^, PO_4_^3−^, ClO_4_^−^, etc., in aqueous solution through a turn-off response [135,136]. The highly toxic CrO_4_^2−^ and Cr_2_O_7_^2−^ anions found in wastewater can be efficiently detected by MOFs, such as [Ln_4_(OH)_4_(bpdc)_3_(bpdca)_0_._5_(H_2_O)_6_](ClO_4_) (Ln = Tb, Gd, bpdca^2−^ = biphenyl-4,4′-dicarboxylate), and [Cd(tptz)(H_2_O)_2_(HCOOH)(ipa)_2_] (tptz = {4-[4-(1*H*-1,2,4-triazol-1-yl)phenyl]phenyl}-1*H*-1,2,4-triazole, ipa = isophthalic acid) [137,138]. The cationic MOF [CuL_2_(H_2_O)_0_._5_](NO_3_)_2_ displays characteristic colors in response to specific anions, such as Cl^−^, Br^−^, I^−^, SCN^−^, and N_3_^−^ (Figure 4) [139]. The isostructural heterometallic MOFs, In/Eu-CBDA, and In/Tb-CBDA (CBDA = 5,5′-(carboxylbis(azanediyl))-diisophthalic acid) can detect 1,4-dinitrobenzene and Cr_2_O_7_^2−^ with high selectivity and sensitivity [140]. ZnO quantum dots on MOF-5 is an effective fluorescent sensing platform for the phosphates tested for the assessment of phosphates in environmental aqueous samples [141], and CdSe/CdS/Cd_0_._5_Zn_0_._5_S/ZnS quantum dots on MOF-5, QD@MOF-5 composite display size-selective thiol sensing [142].

MOF-based sensors for humidity measurements have been studied based on changes of fluorescence or electrochemical signals, such as CuMOF, thin-film of HKUST-1, Cu-BTC film, and amine-functionalized MOF nanoparticles NH_2_-MIL-125(Ti) [143,144,145,146,147]. pH and temperature sensors based on luminescent MOFs have been extensively studied for monitoring pH changes in biological environments and for luminescent thermometers. For example, among others, [Eu_3_(C_14_H_6_N_2_O_4_)_4_(OH)(H_2_O)_4_]·2H_2_O displays a linear photoluminescence response in the 5–7.5 pH range, UiO-66-N=N-ind_3h_ synthesized by post-synthetic modification of UiO-66-NH_2_ exhibits pH-dependent fluorescence in the 1–12 pH range, (Tb-THBA) nanoparticles (THBA = tris[(2-hydroxy-benzoyl)-2-aminoethyl]amine) show a temperature-dependent luminescent intensity in the range of 20–65 °C, and [Zn_3_(TDPAT)(H_2_O)_3_] (TDPAT = 2,4,6-tris(3,5-dicarboxylphenylamino)-1,3,5-triazine) can detect a temperature from 164 to 276 K [148,149,150,151].

Core-shell nanocomposites with an MOF core have been developed for sensing biological molecules, such as human serum albumin, bacterial endospores, and cancer cell apoptosis [152,153,154]. Luminescent MOFs are also successful in detecting DNA, RNA, protein, and other biomolecules and present advantages over other sensing materials for biomolecules (e.g., single-walled carbon nanotubes, graphene oxide, carbon nanoparticles, gold nanoparticles), such as structural diversity, high sensitivity, and biodegradability. Biocompatibility and non-toxic metal clusters need to be developed in order for in vivo sensing to be realized [155].

MOFs and their derived materials are suitable for the construction of electrochemical sensors. The water stable Cu MOF, [Cu_2_(HL)_2_(OH)_2_(H_2_O)_5_]·H_2_O (H_2_L = 2,5-dicarboxylic acid-3,4-ethylene dioxythiophene) was used to construct an electrochemical sensor for simultaneous detection of ascorbic acid and L-tryptophan [156]. Composites containing carbon spheres and Al-MIL-53-(OH)_2_ MOFs on the nafion polymer were used to modify the glassy carbon electrode for the construction of a dopamine sensor. The dopamine signals were enhanced due to the good electrical conductivity and the large surface area of the MOF nanocomposite and the film-forming ability of nafion [157]. Cu-BTC MOFs electrodeposited onto a glassy carbon electrode and modified by graphene oxide were used to construct an electrochemical sensing platform for 2,4,6-trinitrophenol. The sensor can detect TNP in the presence of other nitrophenols due to the high electrical conductivity and high electrocatalytic activity of the nanocomposite [158]. The first example of an electrochemiluminescence(ECL)-active Ru/Zn MOF shows high stability and high ECL due to the large electron transfer of the reaction system, and was used to construct an ECL sensor for cocaine in the serum sample [159]. A turn-on ECL immunosensor for the detection of N-terminal pro-B-type natriuretic peptide (NT-proBNP) was based on MOFs consisting of zinc and tris(4,4′-dicarboxylic acid-2,2′-bipyridyl) ruthenium(II) dichloride combined with the antibodies. The MOFs can enhance the loading of the ECL probe, [Ru(dcbpy)_3_]^2+^, and improve the loading of NT-proBNP-specific antibodies [160]. Recently, field effect transistor (FET) sensors based on MOFs and their derived materials have been developed for practical applications. FET sensors consist of a source and a drain electrode, both of which contact a semiconductor layer. For example, a molecularly imprinted polymer (MIP) film in the presence of MOF-5 was used to construct an FET sensor for the detection of recombinant human neutrophil gelatinase-associated lipid calin [161]. Quartz crystal microbalance (QCM) sensors and piezoelectric sensors based on MOFs have been also developed for detection of small organic molecules (e.g., MeOH, EtOH, MeCN, Me_2_CO). For example, KAUST-7 (NbOFFIVE-1-Ni) and KAUST-8 (AlFFIVE-1-Ni) were used for a QCM sensor for SO_2_, a Cu-BTC/polyaniline nanocomposite for a QCM-based hydrogen sensor, and MIL-101(Cr) for a QCM-based pyridine sensor [162,163,164]. The MOF [Mn_5_(NH_2_bdc)_5_(bimb)_5_]·(H_2_O)_0.5_ (NH_2_bdcH_2_ = 2-amino-1,4-benzene dicarboxylic acid, bimb = 4,4′-bis(1-imidazolyl)biphenyl) displays typical ferroelectric behavior, suggesting that MOFs can be potentially applied for the construction of piezoelectric sensors [165].

### 3.3. Catalytic Applications

**Figure 5:**
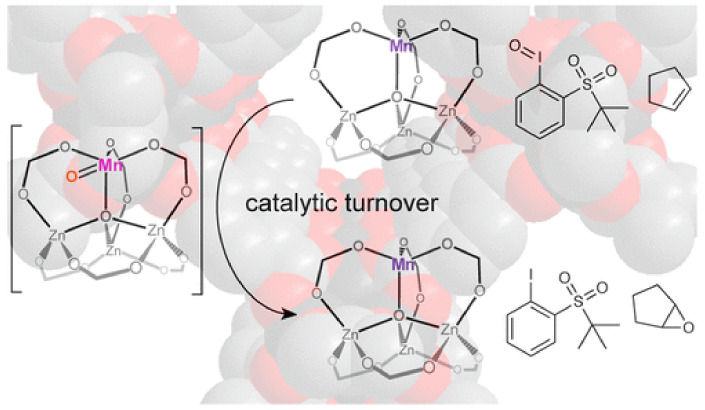
Schematic view of the selective catalytic activity of MnFe-MOF-74 for the epoxidation of the cyclic alkenes in the presence of *^t^*BuSO_2_PhIO. Reprinted with permission from ACS Catal., 2018, 8, 596–601 ([166]). Copyright 2018 American Chemical Society.

MOFs have been extensively used as heterogeneous catalysts for the synthesis of fine chemicals, which are extremely important in the chemical industry. The properties that render MOFs suitable for heterogeneous catalysts are related to the robust nature, which is required for catalysis under extreme conditions, the porosity, and large surface area, which facilitated the catalytic activity as well as the presence of pores and channels, which are needed for catalytic selectivity and the organic ligands that can tune the catalytic reactivity and selectivity. The catalytic active sites of MOFs may be the metal nodes, the functionalized ligands, and the pores of the structure. The synthesis of fine chemicals is most commonly realized through oxidation reactions (e.g., epoxidation, sulfoxidation, aerobic oxidation), 1,3-cycloaddition reactions, transesterification reactions, C–C bond formation reactions (e.g., Heck reaction, Sonogashira coupling, and Suzuki coupling), and hydrogenation reactions of unsaturated organic molecules. The MOFs as heterogeneous catalysts may act as Lewis acids through the metal ions or metal nodes as well as the organic ligands, or as support for the moieties that carry the oxygen or the noble metals necessary for the catalytic reaction. The Zn MOF-5 was partially substituted with manganese and the bimetallic MnFe-MOF-74 was used for the epoxidation of the alkene with high selectivity (up to 99%, Figure 5) [166,167]. Composites of metal complexes immobilized on MOF can also act as Lewis acids in epoxidation reactions, such as post synthetically modified (Cr)NH_2_-MIL-101, post synthetically modified UiO-66 and UiO-67 with salicylaldehyde molybdenum complex, and copper functionalized UiO-66 [168,169,170]. The aerobic oxidation of alcohols to aldehydes or ketones requires the presence of noble metals inside the pores of the MOF or attachment to the modified ligands. Palladium and gold nanoparticles introduced into the nanoporous MOF are used for selective aerobic oxidation [171,172]. Cu-based MOFs are usually used as catalysts for 1,3-dipolar cycloaddition reactions, which is the formation of five-membered ring compounds [173,174]. Several examples of MOF catalysts in transesterification reactions have been reported, such as UiO-66 and UiO-67 [175,176]. C-C bond formation reactions such as Heck reactions, Sonogashira coupling, and Suzuki coupling are extremely important for organic synthesis and require the presence of palladium or palladium nanoparticles as catalysts, which are incorporated in the pores or are attached to the functionalized organic ligands. For example, palladium complexes, such as bis(tri(1-piperidinyl)phosphine) palladium chloride or bis(triphenylphosphine) palladium dichloride incorporated in a Ni-MOF for the Heck reaction of estragole with iodobenzene [177], palladium incorporated in a Zr-MOF based on 2,2′-bipyridine-5,5′-dicarboxylate ligands applied in the carbonylative Sonogashira coupling at atmospheric pressure in the presence of CO [178], and palladium dichloride immobilized on a mixed-ligand MOF containing bipyridyl and biphenyl moieties for Suzuki catalysis [179]. Palladium nanoparticles incorporated in Zr MOF-808 is an excellent heterogeneous catalyst for Heck reaction without an additional base [180], whereas palladium nanoclusters in NH_2_-UiO-66 (Zr) used in the Suzuki catalysis in the presence of light give 99% conversion and selectivity of biphenyl compounds [181]. A wide range of unsaturated organic compounds, such as α,β-unsaturated aldehydes, cinnamaldehyde, nitroarene, and nitro compounds, alkenes and alkynes, quinoline, benzene, and other aromatic compounds, can be hydrogenated with a very high yield and selectivity under mild conditions in the presence of MOFs and derived materials as heterogeneous catalysts. For example, Pt nanoparticles incorporated within MIL-101(Fe,Cr) used as catalysts for the hydrogenation of α,β-unsaturated aldehydes to unsaturated alcohols [182]. MIL-120 incorporated with Ni particles showed a better result on gas-phase benzene hydrogenation than the Ni/Al_2_O_3_ catalyst [183], which is a well-defined hollow Zn/Co ZIF composite with rhombic dodecahedron shape that displayed superior activity and selectivity toward the semi-hydrogenation of acetylene [184], and Ir nanoparticles encapsulated in ZIF-8 used in the hydrogenation of phenylacetylene [185]. The catalytic activity for the CO_2_→CO reduction with [Ru_3_(btc)_2-x_(pydc)_x_X_y_] catalysts (X = Cl, OH, OAc, *x* = 0.1, 0.2, 0.6, 1.0; 0 ≤ *y* ≤ 1.5, H_3_btc = benzene-1,3,5-tricarboxylic acid, H_2_pydc = pyridine-3,5-dicarboxylic acid) as monitored by UHV-FTIR spectroscopy, showed peaks characteristic of the presence of (CO)Ru^δ+^ species. The CO_2_→CO conversion at 90 K is attributed to charge transfer from the 3d Ru orbitals to the 2π_u_ CO_2_ antibonding orbital, possibly yielding chemisorbed CO_2_^δ−^ species that might act as a reaction intermediate to produce CO [111]. These defect-engineered MOFs also act as olefin hydrogenation catalysts after activation with H_2_ to produce Ru-H species, assisted by the presence of the basic pyridyl-N atom of the pydc linkers [111]. Cu-based MOFs, [Cu_3_(btc)_2_] HKUST-1 (btc^3−^ = benzene-1,3,5-tricarboxylate) and [Cu_3_(btb)_2_] MOF-14 (btb^3−^ = benzene-1,3,5-tribenzoate) display high catalytic activity toward CO oxidation at low temperatures (105 K), which is related to the CO species adsorbed on the Cu^2+^ coordinatively unsaturated metal ion sites upon exposure to various amounts of O_2_ [186]. Several MOFs, for example NU-1000, UiO-66, HKUST-1, and MIL-101(Cr)-DAAP, have been tested as heterogeneous catalysts for the catalytic destruction of the phosphate ester bonds and phosphate-fluoride bonds, in chemical warfare agents, such as DMNP (dimethyl 4-nitrophenyl phosphate), DENP (diethyl 4-nitrophenyl phosphate), BNPP (bis(4-nitrophenyl) phosphate), and the highly toxic GD (O-pinacolyl methylphosphonofluoridate), known as Soman [187].

**Figure 6:**
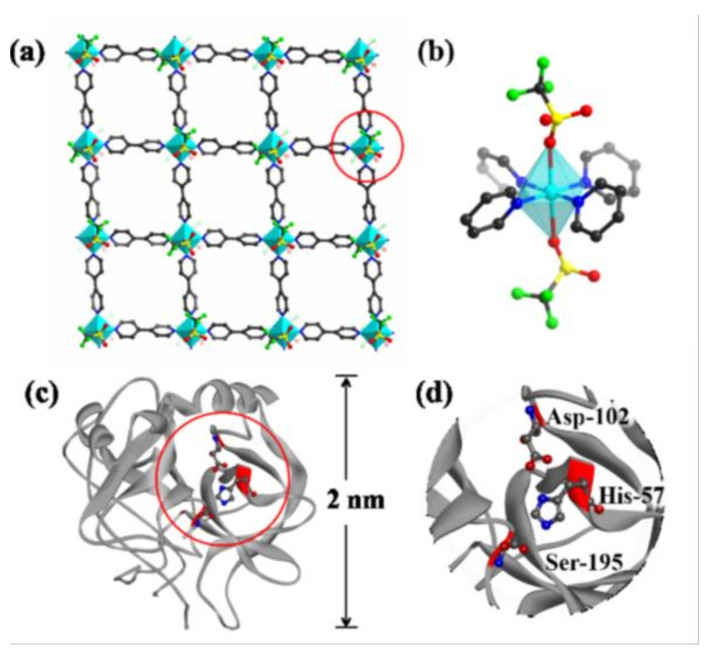
(**a**) Schematic representation of a single-layered 2D nanosheet of Cu-MOF. Color code: C, gray. O, red. S, yellow. F, green. Cu polyhedral, blue. H-atoms not shown. (**b**) Schematic representation of a Cu(II) center in the nanosheets. (**c**) Molecular structure of *a*-chymotrypsin. (**d**) The polychrome sections in this structure are the active site with residues Asp-102, His-57, and Ser-195. *a*-chymotrypsin can be effectively inhibited by 2D Cu-MOF, [Cu(bpy)OTf)_2_] (bpy = 4,4′-bipyridine, OTf = trifluoromethanesulfonate). Reprinted with permission from J. Am. Chem. Soc., 2017, 139, 8312–8319 ([189]). Copyright 2018 American Chemical Society.

Two-dimensional MOFs have been recently developed as catalysts of outstanding intrinsic reactivity, as support materials for catalysts, and as catalysts with multifunctional catalytic activity for diverse organic transformations. Their enhanced catalytic activity is associated with the ultra-thin thickness and more accessible active sites, which decrease the diffusion resistance and increase the host-guest interactions, rendering these materials much better than the corresponding bulk MOFs [188]. For example, 2D MOFs based on tetrakis(4-carboxyphenyl)-porphyrin display unique photochemistry and high efficiency in light-harvesting applications and showed catalytic activity in photooxidation reactions (Figure 6) [189,190,191]. Incorporation of nanoparticles or enzymes as well as post-synthetic modification provided new materials with enhanced catalytic activities [192]. For example, [Zr_12_O_8_(OH)_14_(BPYDC)_9_] (H_2_BPYDC = 2,2′-bipyridine-5,5′-dicarboxylic acid), MON-19, loaded with platinum nanoparticles, displays efficient hydrogenation of C=C bonds under mild conditions without external high-pressure hydrogen [193].

The electrocatalytic activity of MOFs has been investigated in the field of hydrogen evolution reaction (HER), oxygen evolution reaction (OER), oxygen reduction reaction (ORR), carbon dioxide reduction reaction (CO_2_RR), and electrochemical sensing [194]. The requirements for MOFs to display electrocatalytic activity is to possess three electrochemical factors, i.e., onset potential, current density, and redox-active metal sites. MOF-derived electrocatalysts for HER have been extensively studied, such as a bimetallic NiMo-MOF composite with current density of 10 mA·cm^−2^ at low overpotential of 80 mV and Tafel slope of 98.9 mV·dec^−1^, whose enhanced HER activity is due to the structural merits of MOF and the synergy between the MOF and the Ni/Mo metal atoms [195,196]. A cobalt phosphide 2D-MOF nanosheet showed excellent electrocatalytic performance for water splitting, i.e., HER and OER, in acidic and alkaline media with Tafel slopes of 59 and 64 mV·dec^−1^ and a current density of 10 mA·cm^−2^ at the overpotentials of 140 and 292 mV, respectively, which are comparable to those of commercial noble-metal catalysts [197]. Other examples for OER and ORR are a composite of NiCo/Fe_3_O_4_ hetero-particles within MOF-74 with a Tafel slope of 29 mV·dec^−1^ and a current density of 10 mA cm^−2^ at the overpotentials of 238 mV [198], CoNi_2_-MOF (GTGU-10c2) nanobelts with small Tafel slope of 58 mV·dec^−1^ and a current density of 10 mA·cm^−2^ at the overpotentials of 240 mV with long-term stability of more than 50 h in alkaline medium [199], a Co-MOF with a high turn-over frequency of 93.21 s^−1^ at the overpotential of 350 mV and current density of 10 mA·cm^−2^ compared to RuO_2_ [200], and bimetallic Ni/Zn-MOFs with electrocatalytic performance increasing at the higher Ni ratio samples [201]. Recently, Cu-MOF, Zn-BTC-MOF, and Cu-HKUST have been reported for electrochemical reduction of CO_2_ in a standard three electrode set-up for ionic liquids [202,203,204,205].

MOFs have been extensively studied as potential photocatalysts due to their porous nanostructures and controllable semiconductor properties as well as their ability to incorporate co-catalysts such as metals and metal oxides. Their photocatalytic activity is realized through different mechanisms involving parts of the MOFs that absorb light, such as ligand-to-metal charge-transfer mechanism (LMCT), ligand-to-ligand charge transfer mechanism (LLCT), metal-to-ligand charge transfer (MLCT), or metal-to-metal charge transfer (MMCT) mechanism, and dual excitation pathways. For example, [Zr_6_O_4_(OH)_4_L_6_] (H_2_L = 2,2′-diamino-4,4′-stilbenedi-carboxylic acid) was examined for the CO_2_→CO photocatalytic reduction and displays a narrow band gap that absorbs in the visible region with a formation rate of 96.2 μmol·h^−1^·mmol^−1^_MOF_ through an LMCT mechanism [206]. [Zr_6_O_4_(OH)_4_L_6_] (H_2_L = 4,4′-(anthracene-9,10-diylbis(ethyne-2,1-diyl))dibenzoic acid), NNU-28, displays high efficiency for visible-light-driven CO_2_ reduction with a formate formation rate of 183.3 μmol·h^−1^·mmol^−1^_MOF_ through dual excitation pathways involving both the Zr_6_ oxo cluster and the anthracene-based ligand [207]. Encapsulation of the photosensitizer [Ru(bpy)_3_]^2+^ into the porous structure of PCN-99, an indium anionic MOF with H_3_DCTA = 10,15-dihydro-5*H*-diindolo-[3,2-*a*:3′,2′-*c*]carbazole-3,8,13-tricarboxylate acid, Ru(bpy)_3_@PCN-99, displays heterogeneous photocatalytic activity toward the aerobic hydroxylation of arylboronic acid, through the MLCT or MMCT mechanism. The photosensitizer absorbs light and emits an electron, which migrates to the LUMO of the organic ligand or the metal node of the MOF [208]. Graphene oxide (GO)-MOFs composites have been examined as photocatalysts in water-oxidation reactions. The GO-MIL-LIC-1(Eu) composite, in the presence of [Ru(bpy)_3_Cl_2_] sensitizer and Na_2_S_2_O_8_ electron acceptor, under nitrogen atmosphere and visible-light irradiation, displays O_2_ production of 125 μmol, which is more than two times that of the MIL-LIC-1(Eu) MOF [209]. Encapsulation of perovskite quantum dots, CH_3_NH_3_PbI_3_ (MAPbI_3_) in the pores of a Fe-porphyrin MOF PCN-221(Fe_x_), gave the composite photocatalyst MAPbI_3_@PCN-(Fe_0_._2_), which exhibits remarkably high total yield of 1559 μmol·g^−1^ for photocatalytic CO_2_ reduction to CO (34%) and CH_4_ (66%), which is 38 times higher than that of the parent MOF, due to transfer of the photogenerated electrons in the quantum dots to the Fe catalytic sites of the MOF [210]. Core-shell HKUST-1@TiO_2_ composite shows photocatalytic reduction efficiency of CO_2_ to CH_4_ (five times over that of TiO_2_) and selectivity over hydrogen in the photocatalytic reduction compared to parent HKUST-1 and TiO_2_ [211]. Quantum dots nanoparticles in MOFs have been extensively studied as potential photocatalysts, such as the CdS/UiO-66-NH_2_ composite for the selective visible light oxidation of benzyl alcohol to benzaldehyde with molecular oxygen as an oxidant [212], CdS@MIL-101(Fe) nanocomposites for the selective oxidation of benzyl alcohol to benzaldehyde using visible light under mild conditions [213], and CdS/Zn-MOF composites for the photocatalytic water splitting under visible light irradiation [214]. MOFs and their composites, especially the environmentally-friendly Fe-MOFs, are used in advanced oxidation processes (AOPs) as photocatalysts for the removal of organic compounds from water and wastewater by oxidation through reactions with hydroxyl radicals [215,216]. For example, Fe-MOFs with 1,4-piperazinediylbis(methylene) phosphonic acid, STA-12(Fe), used for H_2_O_2_ activation under natural sun-light irradiation, displays highly efficient photocatalytic decomposition of organic dyes from aqueous solution, and demonstrates excellent reusability, suggesting potential application in water depollution [217]. MOFs as heterogeneous photocalysts for chemical warfare agents destruction have been examined, such as Zr-based MOFs (PCN-57 analogues) with benzothiadiazole and benzoselenadiazole, which display selective photocatalytic activity for the oxidation of the mustard gas simulant, 2-chloroethyl ethyl sulfide (CEES) to the nontoxic 2-chloroethyl ethyl sulfoxide (CEESO) [218], and post-synthetically modified Zr_6_-based MOF, NU-1000, with the photosensitizer BODIPY (boron-dipyrromethene) ligand, which shows enhanced singlet oxygen generation for selective detoxification of the sulfur mustard simulant CEES to CEESO with a half-life of ~2 min [219].

### 3.4. Piezo/Ferroelectric, Thermoelectric, and Dielectric Applications

**Figure 7:**
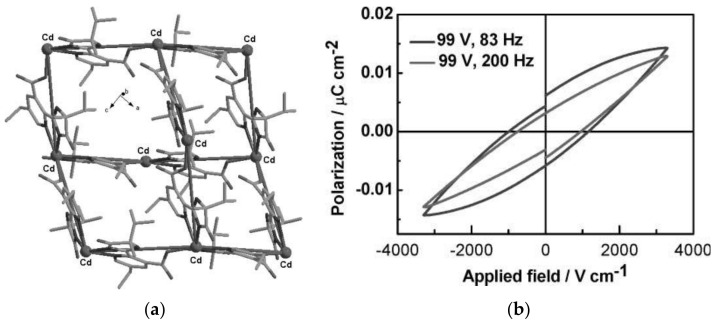
(**a**) The diamond-like net of MOF based on [Cd(imazethpyr)] and (**b**) electric hysteresis loop of the MOF. Dalton Trans. 2008, 3946–3948 ([223])—Reproduced by permission of The Royal Society of Chemistry.

The piezoelectric materials convert mechanical energy into electrical energy through the direct piezoelectric effect and can be considered as energy harvesters to generate energy when direct electricity or batteries are not available. A subclass are the ferroelectric materials, which exhibit spontaneous electric polarization whose direction can be reversed by applying external electric fields. Piezo/ferroelectric materials, such as crystalline and ceramic materials, polymers, and liquid crystals find potential applications in piezoelectric quartz crystals as ultrasonic transducer, sensors and actuators, filters, ultrasonic motors, energy harvester, optical devices and so on. Besides traditional piezo/ferroelectric materials, MOFs have investigated for potential applications, among these [Zn_2_(mtz)(nic)_2_(OH)]·0.5H_2_O (Hmtz = 5-methyltetrazole, Hnic = nicotinic acid), [Zn(phtz)(nic)]_2_ (Hphtz = 5-phenyltetrazole), [Cd(tib)(p-BDC-OH)]·H_2_O (tib = 1,3,5-tris(1-imidazolyl)benzene, p-BDC-OH = 2-OH-1,4-benzenedicarboxylic acid), [In(C_16_H_11_N_2_O_8_]·1.5H_2_O, and [Mn_5_(NH_2_bdc)_5_(bimb)_5_]·0.5H_2_O (NH_2_bdcH_2_ = 2-amino-1,4-benzene dicarboxylic acid, bimb = 4,4′-bis(1-imidazolyl)biphenyl) show significant ferroelectric properties with spontaneous polarization values of P_s_ of 6.26, 5.27, 11.65, 3.81, and 2.556 μC·cm^−2^, respectively [165,220,221,222]. The piezoelectric properties of MOFs have been rarely studied. For example, a MOF based on [Cd(imazethpyr)] displays a piezoelectric coefficient d_33_ value of 60.10 pC·N^−1^, which is smaller than that of BaTiO_3_, but was the first MOF with such piezo/ferroelectricity (Figure 7) [223]. ZIF-8-based MOFs exhibit ‘soft’ piezo/ferroelectricity and water soluble MOFs such as NUS-series and UiO-series exhibit piezoelectric coefficient as d_zz_ up to 3.5 pm·V^−1^ [224,225,226].

The thermoelectric materials, which can generate electric potential from a temperature difference, constitute an environmentally-friendly approach of energy generation from waste heat. Besides inorganic compounds, such as oxides and alloys, the approach of conductive MOFs as new potential thermoelectric materials has been developed. This approach includes first-row transition metal MOFs with thiolate ligands, such as [Cu(pdt)_2_] (pdt = 2,3-pyrazinedithiolate), inclusion of guest molecules in known MOFs, such as TCNQ@HKUST-1 (HKUST-1 = [Cu_3_(BTC)_2_], BTC = benzene tricarboxylate), I_2_ and metal nanoclusters in order to improve the conductivity of the material. 2D MOF nanosheets of bis(thiolato) ligands and light transition metals, i.e., π-d conjugated systems, and post-synthetically modified MOFS, i.e., guest@MOFs and conductive-polymer grafted MOFs, are promising candidates for the fabrication of thermoelectric devices due to their excellent conductivity [227].

Semiconducting devices are based on dielectric materials, which display ultra-low dielectric constants (*κ* < 3.9 as in SiO_2_). MOFs feature ultra-low dielectric constants, which are considered as promising materials for the future microelectronics industry. The requirements of doing so are thermal stability at a high temperature, predictable mechanical behavior, electrical insulation, and adhesion to other interlayers. DFT calculation on various MOFs, such as IRMOF-1 family, UiO-66, UiO-67, MIL-140, and MOF-74-M (M = Mg, Mn, Fe, Co, Ni, Zn), revealed the influence of the structural and chemical characteristics on their electronic and dielectric properties, demonstrating their ability to behave as insulators and low-dielectric constant materials, and predicted dielectric constants in the range of 1.25 to 2.0 [228,229]. Surface-anchored HKUST-1 thin films grown by liquid phase epitaxy (LPE) were studied by spectroscopic ellipsometry (SE) to determine an optical constant of *n* = 1.39 at a wavelength of 750 nm (*κ*~1.93) [230]. ZIF-8 thin films deposited on silicon wafers studied by SE and the dielectric constant was measured by impedance analysis at different frequencies and temperatures yielding *κ* = 2.33 at 100 kHz [231]. Other MOFs, which display ultra-low dielectric constants (below that of SiO_2_) are, for example, [Sr_2_(1,3-dbc)_2_(H_2_O)_2_] (bdc = 1,3-bis(4,5-dihydro-2-oxazolyl)benzene), which retains its crystallinity up to 420 °C with *κ*~2.4 [232], [Zn_2_(Hbbim)_2_(bbim)] (H_2_bbim = bisbenzimidazole) with *κ*~3.05 [233], [Pb(tab)_2_(4,4′-bipy)](PF_6_) (tab = 4-(trimethylammonio)benzenethiolate) with *κ*~2.53, [Zn_2_(L-trp)_2_(bpe)_2_(H_2_O)_2_] (L-trp = L-tryptophane, bpe = 1,2-bis(4-pyridyl)ethylene) with *κ*~2.53, [Mn_2_(D-cam)_2_(2-Hpao)_4_] (D-cam = D-camphoric acid, 2-Hpao = 2-pyridinealdoxime) with *κ*~2.8, and [Co_2_(D-cam)_2_(3-abpt)_2_(H_2_O)_3_] (3-abpt = 4-amino-3,5-bis(3-pyridyl)-1,2,4-triazole) with *κ*~3.0 [234].

### 3.5. Proton-Conducting and Magnetic Materials

**Figure 8:**
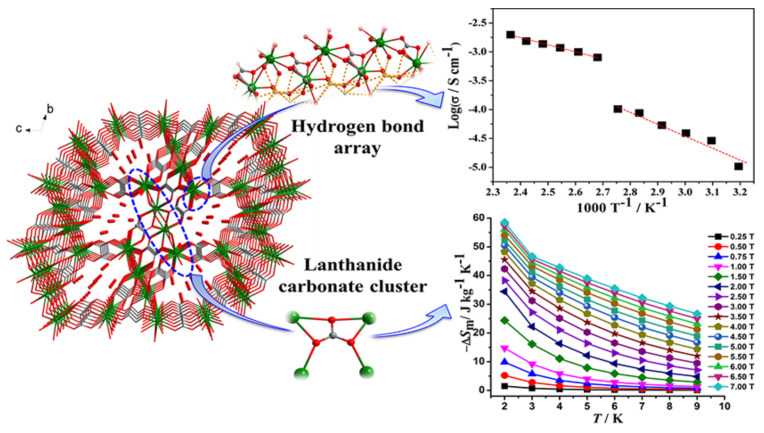
The 3D framework of {[Gd_2_(CO_3_)(ox)_2_(H_2_O)_2_]·3H_2_O, which contains an ordered one-dimensional pore channel along the a-axis, which serves as a 1D hydrogen bond pathway. The compound shows high proton conductivity (up right) and a large magnetocaloric effect (down right). Reprinted with permission from Inorg. Chem. 2018, 57, 9020–9027 ([239]). Copyright 2018 American Chemical Society.

Recently, MOFs have attracted considerable interest as proton-conducting materials and their potential applications in electrochemical devices, sensors, hydrogen fuel cells, etc. MOFs can accommodate various proton carriers in their pores/channels and can provide useful insight into the proton-conducting pathway and mechanism, and these advantages render them excellent materials for such applications. While the negative charge of the lattice is not a prerequisite for conducting protons, many of them are anionic MOFs whose charge is offset by small protonated amines or protons attached to the solvent molecules by hydrogen bonds, or protons weakly attached to functional groups of the ligands (less frequently in carboxylates, more often in sulfonic and phosphonate ligands). For example, {H[(N(CH_3_)_4_)_2_][Gd_3_(NIPA)_6_]}·3H_2_O (H_2_NIPA = 5-nitroisophthalic acid) displays high proton conductivity of 7.17 × 10^−2^ S·cm^−1^ at high relative humidity, which is among the highest values for proton-conducting MOFs [235], and the bimetallic complex {NH(prol)_3_}[MnCr(ox)_3_] (NH(prol)_3_^+^ = tri(3-hydroxypropyl)ammonium, ox = oxalate), which consists of oxalate-bridged bimetallic layers interleaved by NH(prol)_3_^+^ ions and shows proton conduction of ~10^−4^ S·cm^−1^ under 75% relative humidity due to the presence of an extensive H-bonded network between the anionic MOF, the NH(prol)_3_^+^ ions, and water molecules [236]. Some examples of neutral MOFs, which display high proton conductivity, are [Tb_4_(TTHA)_2_(H_2_O)_4_]·7H_2_O (H_6_TTHA = 1,3,5-triazine-2,4,6-triamine hexaacetic acid) with high proton conductivity over 10^−2^ S·cm^−1^ at 295–358 K temperature range due to an extensive H-bond network formed between water solvates and carboxyl groups [237], [Ln(L)(H_2_O)_2_] (Ln^III^ = Dy, Er, Gd; H_3_L = (HO)_2_P(O)CH_2_CO_2_H) with proton conductivity values of 1.13 × 10^−6^, 2.73 × 10^−3^, and 6.27 × 10^−6^ S·cm^−1^, respectively, at high temperatures (>348 K) and 95% relative humidity [238], and [Ln_2_(CO_3_)(ox)_2_(H_2_O)_2_]·3H_2_O (Ln^III^ = Ce, Pr, Nd, Tb) with proton conductivity above 10^−3^ S·cm^−1^ without an additional humidity (Figure 8) [239].

The porous nature of MOFs combined with magnetic properties induced by transition and/or lanthanide metal ions could find interesting applications such as in magnetic sensors. The magnetic properties of MOFs have been well documented and understood [240,241,242]. Metal-radical approaches have been used to synthesize magnetic MOFs such as MOROF-1, [Cu_3_(PTMTC)_2_(py)_6_(EtOH)_2_(H_2_O)] (H_3_PTMTC = perchlorotriarylmethyl tricarboxylic acid radical), which combines very large pores with bulk magnetic ordering [243]. The structural changes during solvent inclusion can be monitored by the magnetic properties. MOROF-1 displays reversible behavior for ethanol and methanol, showing the selectivity of the sponge-like magnetic sensor. Other examples by the same group are a 3D metal-organic open-framework, [Tb(PTMTC)(DMF)_3_], which displays a rare lattice complex *T* structure with large channels, a fully reversible guest-induced reversible crystal to amorphous transformation and ferromagnetic metal-radical interactions [244], and the 3D MOFs [Cu_6_(PTMHC)_2_(4,4′-bipy)_3_(H_2_O)_12_] and [Cu_6_(*a*H-PTMHC)_2_(4,4′-bipy)_3_(EtOH)_6_(H_2_O)_6_], which show metal-radical ferromagnetic and weak antiferromagnetic interactions, respectively [245].

### 3.6. Biomedical Applications

MOFs and their derived materials have been increasingly studied as drug carriers, bioimaging agents, and therapeutic agents due to their excellent physicochemical properties. The majority of known drug carriers, such as liposomes, nanoparticles, and micelles, show poor drug loading (less than 5%) and rapid drug release. Therefore, porous MOFs with high drug loadings are considered as candidates for delivery applications. The requirements for efficient drug carriers are the high load of drugs, the control of the drug release, the control of matrix degradation, and the low toxicity. The loading of the drugs into MOFs can be achieved by non-covalent encapsulation into the MOF by physisorption, by post-synthetic modification of the organic ligands after the synthesis of the MOF, by the use of the drugs as organic ligands in building the MOFs, and by attaching the drugs to the subunits of the MOF [246].

**Figure 9:**
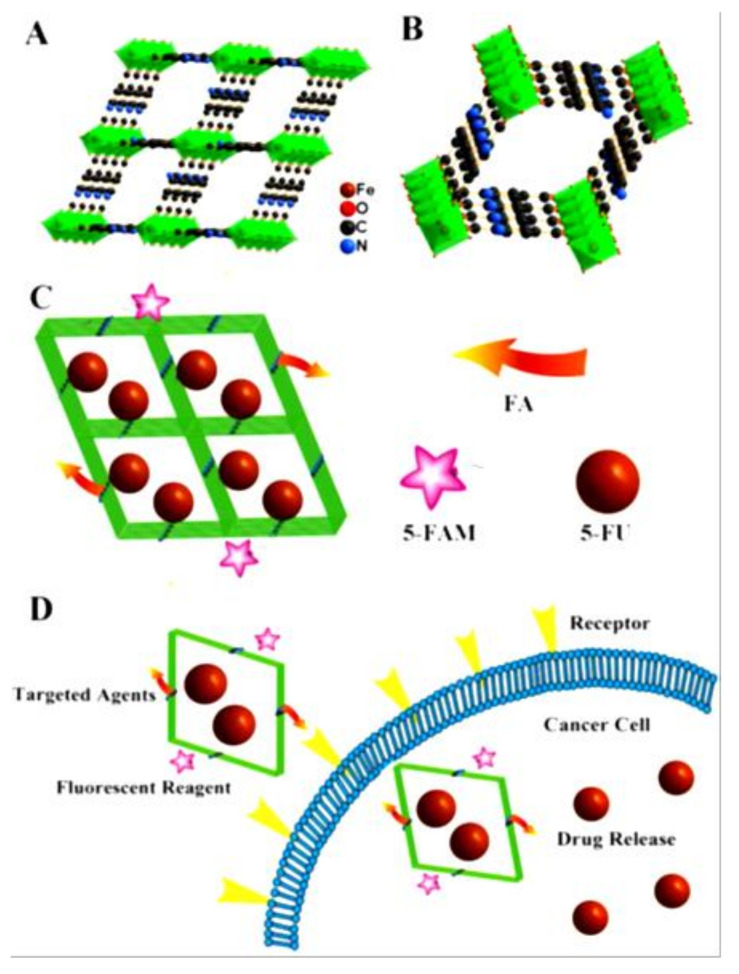
2D plane view of Fe-MIL-53-NH_2_ (**A**,**B**), schematic representation of the drug delivery system Fe-MIL-53-NH_2_-FA-5-FAM/5-FU (**C**), and schematic illustrations of Fe-MIL-53-NH_2_-FA-5-FAM/5-FU for targeting drug delivery (**D**). Reprinted with permission from ACS Appl. Mater. Interfaces 2017, 9, 3455–3462 ([250]). Copyright 2017 American Chemical Society.

MIL-100 and MIL-101 based on trimetallic nodes and BTC (1,3,5-benzene tricarboxylic acid) or BDC (1,4-benzene dicarboxylic acid) were the first MOFs suggested as drug delivery systems in 2006. It was shown that both MIL-100 and MIL-101 are able to absorb very large amounts of ibuprofen (up to 1.4 g per gram of MIL-101), which was completely released under physiological conditions in three (MIL-100) or six (MIL-101) days [247]. [Gd(BDC)_1_._5_(H_2_O)_2_] nanorods and [Gd(1,2,4-BTC)(H_2_O)_3_].H_2_O nanoplates were proposed as nanoscale contrast agents for MRI [248]. Recent developments in anticancer therapy involve the generation of hydroxyl radicals ·OH from H_2_O_2_, catalyzed by Fe^3+^ ions as well as Mn^2+^, Cu^+^, Cr^4+^, according to the Fenton reaction. Reactive oxygen species (ROS), such as O_2_^−^, H_2_O_2_, ·OH, ClO^−^ etc., are produced by the uncontrolled growth of the tumor and the dysfunction in metabolism. The hydroxyl radicals are able to cause damage to tumor cells, which are more than any other ROS. Modern anti-cancer therapies, such as photodynamic therapy (PDT), sonodynamic therapy (SDT), and chemodynamic therapy (CDT) are based on the development of the above studies. MOFs have been applied for PDT, SDT, and CDT since the last decade [249]. An Hf-porphyrin nanoMOF, DBP-UiO (DBP = 5,15-di(p-benzoato)porphyrin), acts as an excellent PDT photosensitizer as indicated from the efficient generation of ^1^O_2_ and its cytotoxic assay [246]. The drug delivery system Fe-MIL-53-NH_2_-FA-5-FAM/5-FU is based on the Fe-MIL-53-NH_2_ MOF, which displays high loading capacity for the anti-cancer drug 5-fluorouracil (5-FU), conjugated with the fluorescence imaging agent 5-carboxyfluorescein (5-FAM) and folic acid (FA), and exhibits better toxicity to cancer cells due to the targeted 5-FU release and acts as a potential contrast agent for MRI (Figure 9) [250]. Nanospheres of [Zn(bix)] (bix = 1,4-bis(imidazole-1-ylmethyl)benzene) can encapsulate and release known anticancer drugs, such as doxorubicin (DOX), camptothecin (CPT), SN-38, and daunomycin (DAU), and show very strong cytotoxic effects against human promyelocytic leukemia cells (HL60) due to the release of DOX from the MOF spheres, causing the death of cancer cells [251].

One of the most important issues for the application of MOFs as potential drug delivery systems is the issue of biotoxicity. That is, MOFs may be harmful to humans. For this reason, biological MOFs based on active pharmaceutical ingredients, such as amino acids, proteins, peptides, and low toxicity metal ions, such as zinc and iron, have been developed (BioMOFs). For example, [Zn(cys)_2_] (cys = cystine) tailor with methylene blue (MB) and sorafenib (SOR) was tested as a drug delivery system against colorectal cancer and Leishmania in PDT (for MB) and hepatocellular carcinoma (for SOR) [252]. Iron-based MOFs, such as MIL-100 and MIL-88A, showed no cytotoxicity to mouse macrophages J774, A1, human leukemia, and human multiple myeloma cells, and low iron concentration in tissues after 1, 7, and 30 days of treatment. Other important issues for potential application as drug delivery issues are the size, shape, and the biological stability of the MOFs [253]. UiO-66 and UiO-67 modified with poly(ε-caprolactone) have been applied as potential drug carriers for the anti-cancer drugs paclitaxel and cisplatin [254]. Fe-MIL-100, Zr-UiO-66, Fe-MIL-53, and Fe-MIL-127 have been used as caffeine carriers [255,256]. Sodium diclofenac was loaded into ZJU-101 through ion exchange and penetration procedures and showed a more quick release of the drug in inflamed tissues with lower pH (5.4) than normal tissues (7.4) [257]. Ion exchange of dimethylammomium cations with procainamide in {[Zn_8_O(ad)_4_(BPDC)_6_]·2Me_2_NH_2_·8DMF·11H_2_O} (ad = adeninate; BPDC = biphenyldicarboxylate), known as bio-MOFs-1, showed drug loading up to 22 wt % after 15 days and slow drug release in pure water [10]. Current trends in nanomedical applications of MOFs in PDT and other anti-cancer treatments involve surface functionalization of the external surface of MOFs in order to fit specific requirements. For example, grafting of functional polymers such as PEG, in order to improve the colloidal stability, tailoring of fluorophore for bioimaging applications, and functionalization of targeting molecules such as peptides for target binding [258]. For example, the azide group in UiO-66-N_3_ can react with the alkane group via click reactions, thus, modifying the target molecule on the surface of the MOF [259]. The coordinative incorporation of oligohistidine-tags on metal-organic framework nanoparticles based on MIL-88A, HKUST-1, and Zr-fum was investigated for the cellular uptake of peptides and proteins with MOF-NPs [260].

MOFs have been investigated as antibacterial and antifungal agents. For example, BioMIL-5 derived from Zn(II) and azelaic acid displays a 3D nonporous framework and shows interesting dermatological and antibacterial effects against the Gram positive bacteria *S.aureus* and *S.epidermidis* [261], [Zn(hzba)_2_]·2.4H_2_O (hzba = 4-hydrazinebenzoate) inhibited the bacterial growth and metabolic activity of *Staphylococcus aureus* [262], Ag(I)-MOFs were tested against *S.aureus* and *E.coli* and showed significant antibacterial activity [263], and the first antibacterial Co-MOF, Co-TDM (TDM = tetrakis[(3,5-dicarboxyphenyl)-oxamethyl]methane) inactivates the Gram negative bacteria *E.coli* [264]. HKUST-1 showed strong anti-fungal activity against *Saccharomyces cerevisiae* and *Geotrichum candidum* due to the release of copper ions into the medium after breaking down the crystal of the MOF [265], and [Cu_3_(BTC)_2_(H_2_O)_3_] (BTC = 1,3,5-benzenetricarboxylate) was investigated against *Aspergillus oryzae*, *Candida albicans*, *Fusarium oxyporum*, and *Aspergillus niger* and exhibited powerful anti-fungal activity due to its ability to reduce the oxygen gas and the production of ROS, which damage the cell and inhibit the microorganisms [266].

### 3.7. Analytical Applications

MOFs have been recently studied as advanced sorbent materials in sample preparation of small organic molecules, such as MIL-53(Al) for polycyclic aromatic hydrocarbons (PAHs) and pharmaceuticals; MIL-101(Cr) for PAHs, bichlorinated biphenyls (PCBs), volatile organic compounds (VOCs), pharmaceuticals, herbicides, and pesticides; IRMOF-3 for PAHs; ZIF-8 for PAHs and VOCs; MOF-5 for PAHs, PCBs, and VOCs; MIL-100(Fe) and UiO-66-NH_2_ for PCBs; UiO-67 for VOCs and pesticides [267]. Solid phase extraction (SPE) of biomacromolecules from biological fluids by using MOFs as sorbents is one of the most efficient methods. Well known and thoroughly studied MOFs and composites, such as MIL-53, MIL-100, MIL-101, Fe_3_O_4_@[Cu_3_(btc)_2_], Fe_3_O_4_@MIL-100/101(Fe), and MIL-101(Cr)-NH_2_, have been successfully used for SPE of low abundance peptides, phosphopeptides, clycopeptides, nucleic acids, and proteins from biological fluids, such as human plasma, urine, serum, saliva, egg white, and milk [268,269]. In addition, MOFs are used as absorbents for sample preparation of food matrices, such as milk, beverages, meat and chicken products, and fish, prior to analysis with chromatographic and spectrometric methods [270].

## 4. Concluding Remarks

Metal-organic frameworks have attracted increased interest due to their specific structural features, related to their porous nature and large specific surface area, as well as high thermal stability. MOFs can be easily synthesized under ambient or extreme conditions at high temperature and high pressure, and also by green chemistry methods, such as by mechanochemical methods and by electrochemical and sonochemical methods. The post-synthetic modification of the MOFs after their synthesis has been widely used to introduce functional groups and give the desired physical and chemical properties. For large-scale commercial production, MOFs are synthesized in continuous-flow solvo(hydro)thermal/tank/microfluidic or milli-fluidic reactors. The possibility of functionalization of the size and shape of the pores offers high potential for applications in the energy and environment, including gas sorption, storage, and separation, as well as metal ions and toxic molecules for analytical and sensing purposes, inclusion of drugs, and biologically important molecules as smart carriers for anti-cancer and anti-bacterial therapies. MOFs can be modified with nanoparticles, polymers, and cyclodextrines to form hybrid composite materials, which have been tested as smart materials for catalysis, drug delivery systems, and new therapeutic agents, textiles for air filters, radiation blocking, and noise reduction, sensors for gases, and ions for separation purposes.

The multifunctional nature of MOFs and their composite materials offers high impact for development of new materials for clean and emerging technologies in the automotive industry, energy production, clean air and water, and health. MOF-based commercial products have been already moved to market from startups in US and Europe for carbon capture, storage of highly toxic gases in the semiconductor industry, capture of water from humid air, selective separation of lithium ions for electric vehicles, removal of toxic metals and ions from water, and adsorbent nanomaterials. MOFs and derived materials are at the ‘heart’ of the smart materials needed to evolve the fourth industrial revolution during our century.

## Data Availability

Data available in a publicly accessible repository.
